# ^68^Ga-DOTATATE PET/CT imaging for insulinoma in MEN1 patient with endogenous hyperinsulinemic hypoglycemia: A case report

**DOI:** 10.1097/MD.0000000000030252

**Published:** 2022-08-26

**Authors:** Yunuan Liu, Xinming Zhao, Jingmian Zhang, Jianfang Wang, Zhaoqi Zhang, Meng Dai, Na Wang, Fenglian Jing, Tingting Wang, Weiwei Tian

**Affiliations:** a Department of Nuclear Medicine, The Fourth Hospital of Hebei Medical University, Shijiazhuang, China.

**Keywords:** ^18^F-FDG, 68Ga-DOTATATE, insulinoma, MEN1, neuroendocrine tumor, PET/CT

## Abstract

Multiple endocrine neoplasia type 1 (MEN1) syndrome is a rare and complicated disease that is associated with several endocrine tumors. Here, we report a case of MEN1 associated with insulinoma, parathyroid, and pituitary tumors by ^68^Ga-DOTATATE positron emission tomography/computed tomography (PET/CT).

**Patient concerns:**

A 49-year-old woman presented with intermittent hypoglycemia for more than a year and developed indistinct consciousness without an apparent trigger.

**Diagnoses:**

Biochemical results showed abnormally high serum insulin and parathyroid hormone levels. She underwent an Abdominal magnetic resonance imaging revealed a small nodule in the uncinate process of the pancreas, but it did not clarify the nature of the small nodule. Pituitary magnetic resonance imaging scan revealed a micropituitary tumor, and parathyroid imaging showed no abnormalities. ^18^F-FDG PET/CT showed no apparent abnormal ^18^F-FDG uptake in the whole body. In contrast, ^68^Ga-DOTATATE PET/CT imaging showed pathological radiotracer uptake in the pancreatic uncinate process, accompanied by mild radiotracer uptake in the pituitary gland, and no apparent abnormal radiotracer uptake in the parathyroid area.

**Interventions:**

The patient underwent echoendoscopy for pancreatic uncinate process lesions and surgical resection.

**Outcomes:**

Histological analysis was suggested of insulinoma of pancreatic neuroendocrine tumor, the Ki-67 index was low (only 1% being positive).

**Lessons:**

This case demonstrates that ^68^Ga-DOTATATE can be used for the detection of MEN1-related tumors and preoperative localization of small and low-grade insulinomas by PET/CT.

## 1. Introduction

Multiple endocrine neoplasia type 1 (MEN1) is a rare hereditary disease with autosomal dominant inheritance. MEN1 is characterized by the development of several endocrine tumors in a single individual; the most common are tumors of the parathyroid gland, pituitary gland, and neuroendocrine tumors (NETs) in the pancreatic islets.^[1,2]^ The diagnosis of MEN1 needs to meet one of the following criteria: the development of 2 or multiple MEN1-related endocrine neoplasms (including intrapancreatic tumor, parathyroidoma, pituitary adenoma, and others), the appearance of MEN1-related neoplasms in first-degree relatives for 1 patient with MEN, and testing for MEN1 gene mutation in a patient who may be asymptomatic and has not yet abnormal findings.^[1]^ Pancreatic insulinoma is a typical functioning pancreatic NET in MEN1 that causes endogenous hyperinsulinemic hypoglycemia.^[3]^

Imaging is of paramount importance for the diagnosis of MEN1-associated tumors. Normally, conventional imaging (such as US, CT, or MR) can offer detailed anatomical features and aggressive expansion of tumor cells; however, the small size of NETs makes it difficult to detect the primary tumors or their metastases using conventional anatomic imaging. Considering that anatomic imaging patterns are not able to describe the peculiar characteristics of endocrine tumors, they suggested that the diagnostic sensitivity and accuracy of functional imaging are better than those of conventional anatomic imaging.^[4–6]^ The combined ^68^Ga-SSA (somatostatin analog)/^18^F-FDG positron emission tomography/computed tomography (PET/CT) imaging has received particular attention because its potential application can reflect the molecular biological characteristics of MEN1-related NETs from the expression level of somatostatin receptor (SSTR) and the level of glucose metabolism, respectively.

Herein, we report a rare case of MEN1 associated with insulinoma due to intermittent hypoglycemia for more than 1 year, and without a family history, which was successfully diagnosed by means of a novel tool of ^68^Ga-DOTATATE PET/CT after negative ^18^F-FDG PET/CT.

## 2. Case report

**Figure 1.**
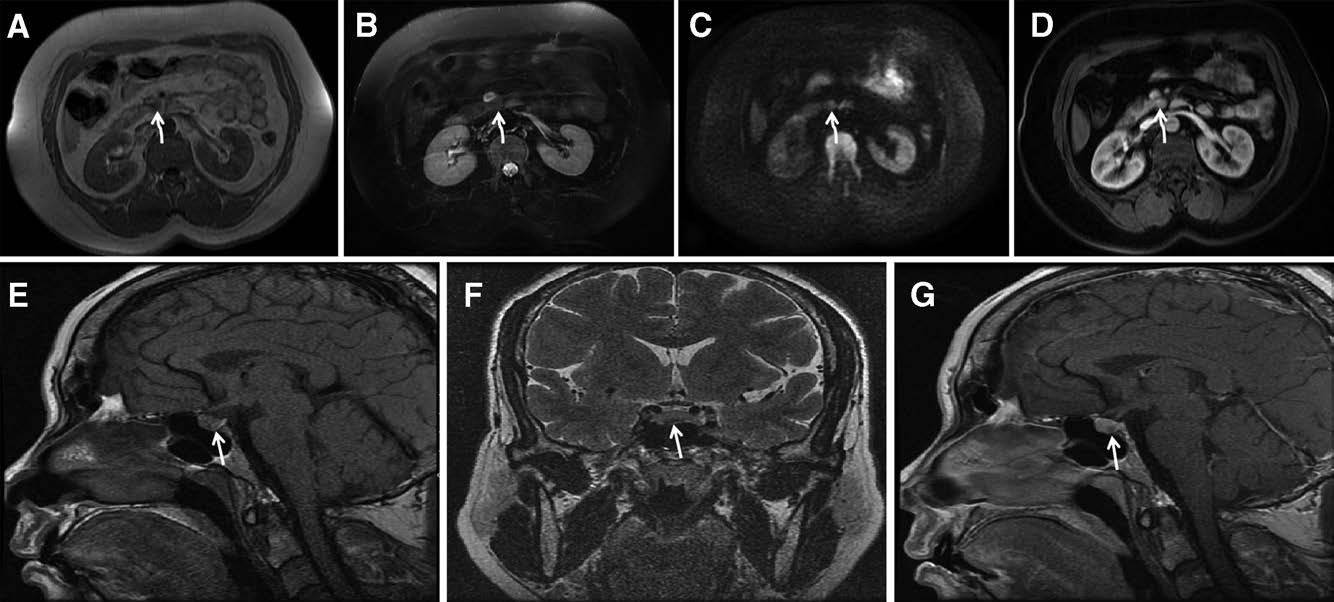
Upper row (A–D): MR study of the uncinate process of the pancreas. Lower row (E–G): MR study of the pituitary. (A) A small nodule with slightly low signal on T1WI. (B) Identical signal on T2WI. (C) Slightly high signal intensity on DWI. (D) The lesion was with minimal enhancement. (E–G) Identical signal on T1WI and slightly low signal on T2 in the right lower pituitary, the enhanced scan revealed a hypointense lesion. MR = magnetic resonance, T1WI = T1-weighted imaging, T2WI = T2-weighted imaging.

A 49-year-old woman presented with intermittent hypoglycemia for more than a year and developed indistinct consciousness without an apparent trigger, which improved after eating. A physical examination revealed no abnormalities. Serum insulin and C-peptide levels (92.2 uIU/mL and 8.34 ng/mL, normal range: 6.00–27.00 uIU/mL and 0.90–7.1 ng/mL) were abnormally high, and serology testing showed fasting serum glucose level (2.02 mmol/L, normal range: 3.90–6.1 mmol/L) was low. The islet cell antibody test result was weakly positive. Serum parathyroid hormone level (204 pg/mL, normal range: 12.00–65.00 mmol/L) was significantly elevated, blood calcium level (2.6 mmol/L, normal range: 2.11–2.52 mmol/L) was modestly elevated, and blood phosphorus level (0.69 mmol/L, normal range: 0.85–1.51 mmol/L) was modestly decreased. The plasma and urinary cortisol levels were normal; hence, adrenal disease was ruled out. Her serum human growth levels were normal. The clinician suspected the presence of insulinoma. Abdominal enhanced magnetic resonance imaging (MRI) (Fig. 1A–D) revealed slightly increased soft tissue in the uncinate process of the pancreas, T1-weighted imaging showed slightly low signal intensity (Fig. 1A, curved arrows), T2-weighted imaging showed identical signal intensity (Fig. 1B, curved arrows), DWI imaging showed slightly high signal intensity (Fig. 1C, curved arrows). Contrast-enhanced MRI (Fig. 1D, curved arrows) showed mild enhancement. Parathyroid imaging showed no abnormalities, while pituitary enhanced MRI revealed a micro-pituitary tumor (9.0 × 3.2 mm, as shown in Fig. 1E–G).

**Figure 2.**
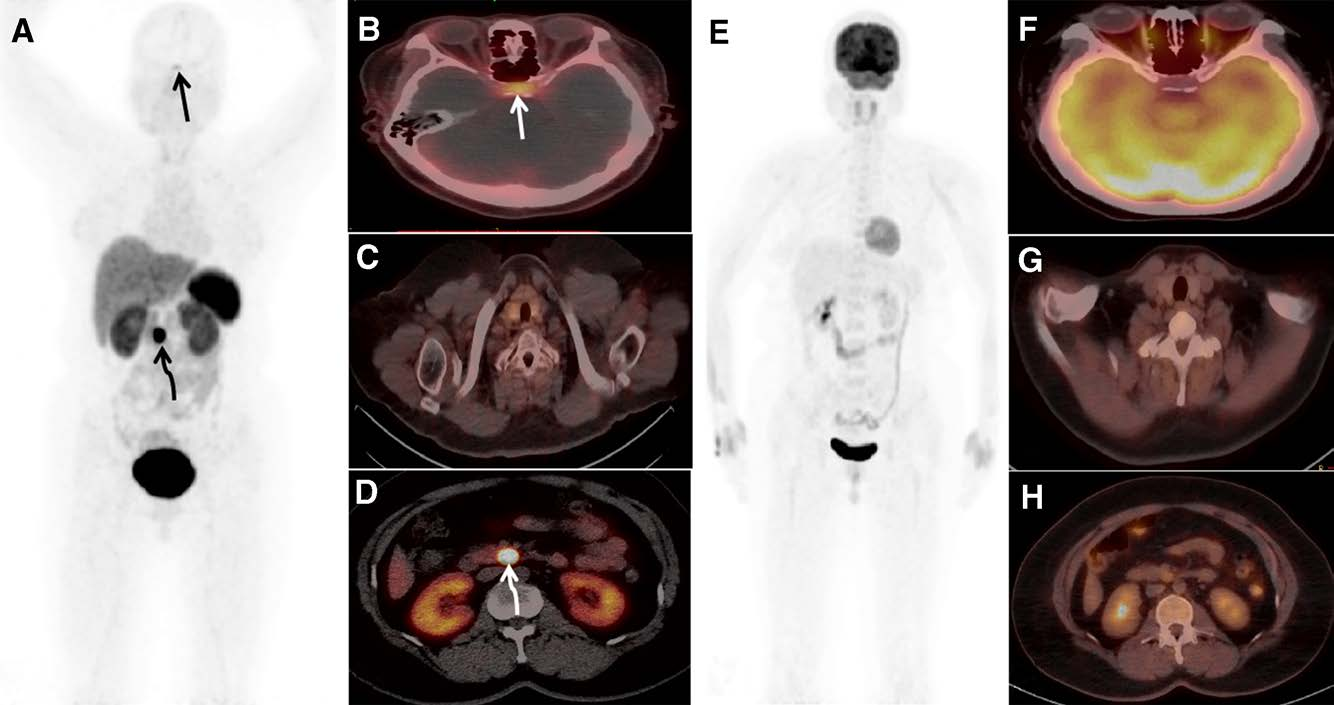
^68^Ga-DOTATATE PET/CT images (A–D) and ^18^F-FDG PET/CT images (E–H). (A) The MIP and axial fusion images of the ^68^Ga-DOTATATE PET/CT showing 1 focal areas of increased radiotracer uptake in the uncinate process of the pancreas (A and D, curved arrows) and a mild area of radiotracer uptake in the pituitary (A and B, thin arrows), whereas the parathyroid region was no significant abnormalities on PET/CT imaging (A and C). The MIP of ^18^F-FDG PET/CT and axial ^18^F-FDG PET/CT fusion images (E-H) showed no obvious abnormal ^18^F-FDG uptake. PET/CT = positron emission tomography/computed tomography.

Therefore, the patient underwent ^18^F-FDG and ^68^Ga-DOTATATE PET/CT scan, the MIP of ^18^F-FDG PET/CT and axial ^18^F-FDG PET/CT fusion images showed no apparent abnormal ^18^F-FDG uptake (Fig. 2E–H). In contrast to ^18^F-FDG PET/CT, the MIP of 68Ga-DOTATATE PET/CT showed pathological radiotracer uptake in the upper abdomen and mild area of radiotracer uptake in the pituitary (Fig. 2A, curved arrows and thin arrows); the axial fusion images of ^68^Ga-DOTATATE PET/CT showed pathological radiotracer uptake in the uncinate process of the pancreas (14 × 12 mm, as shown in Fig. 2A and D, curved arrows), along with mild radiotracer uptake in the pituitary (Fig. 2A and B, thin arrows), whereas the parathyroid region showed no significant abnormalities on ^68^Ga-DOTATATE PET/CT imaging (Fig. 2A and C).

**Figure 3.**
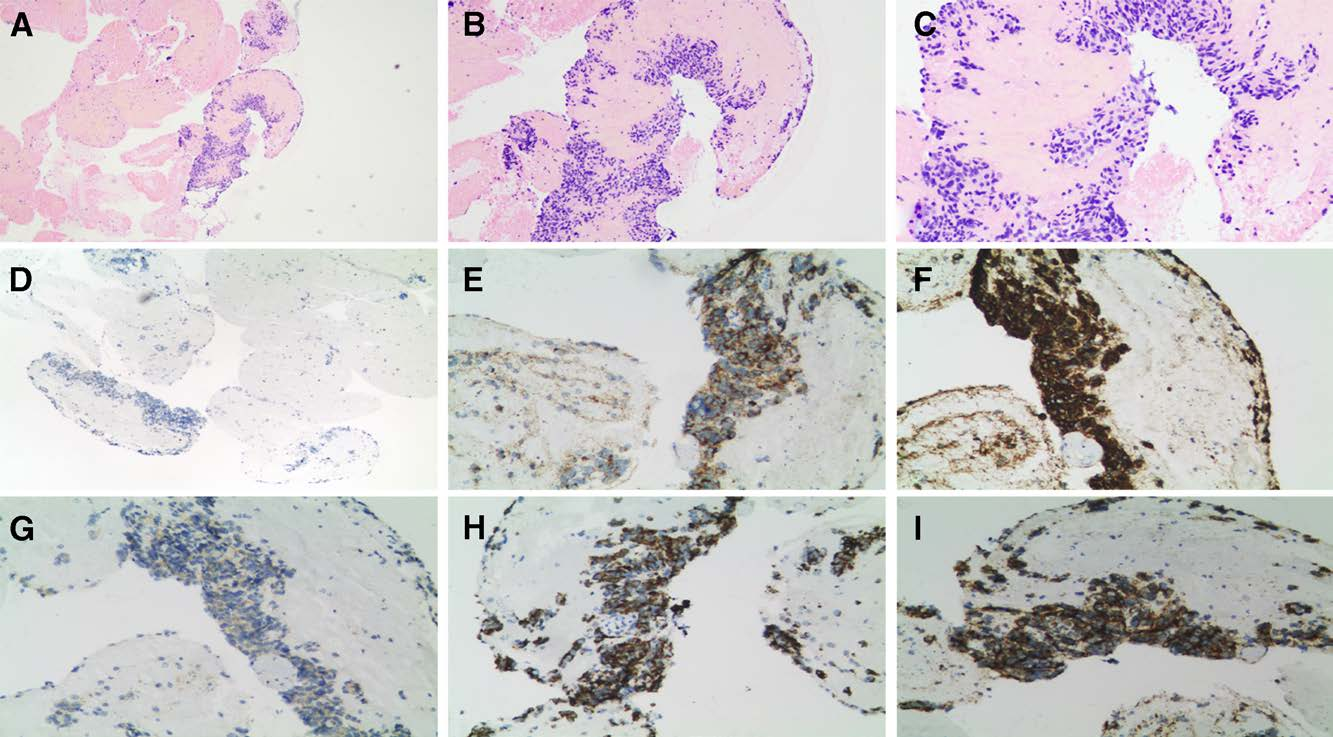
(A–C) The pathological results of pancreatic uncinate lesion showed pancreatic tissue and free ductal epithelial components were seen in the puncture material, and a few clusters of tumor cells were seen next to the adipose tissue (A: 10 × 4 magnification, B: 10 × 10 magnification, C: 10 × 20 magnification). (D–I) The immunohistochemistry of pancreatic uncinate lesion of Ki-67(1%+), CgA(+), Syn(+), CD56(+), CK8/18(+) and β-catenin showed positive staining(10 × 10 magnification).

Subsequently, the patient underwent endoscopic ultrasound-guided fine-needle aspiration biopsy for the pancreatic uncinate process lesion and surgical resection, and the hypoglycemic symptoms disappeared after surgical resection. The pathological slides are shown in Figure 3A–C. Immunohistochemical analysis showed the lesion to be Ki-67(1%+, that is, 1% of the cells were positive for the proliferation marker Ki-67), CgA(+), Syn(+), CD56(+), CK8/18(+), β-catenin(+) (Fig. 3D–I). Histological analysis indicated the pancreatic uncinate process lesion to be a well-differentiated NET, and G1 grade, which was suggestive of insulinoma of the pancreatic NET (pNET), combined with all the relevant biochemical tests.

## 3. Discussion

Insulinomas account for 10% to 30% of pancreatic tumors in MEN1-associated patients; in other words, they can also be said to be insulin-secreting cells that are tumors on β-islet cells.^[1]^ The primary manifestations are insulinomas in 10% of MEN1-associated patients, and there a few insulinoma patients are accompanied by MEN1.^[7]^ The most common types of functioning pNETs are insulinomas, which usually appear as solitary and small tumors. Accurate preoperative localization for insulinomas is widely accepted, since some small insulinomas may not be found during surgery. Nevertheless, preoperative localization of insulinomas is a difficult clinical problem because of their small size and close resemblance to surrounding tissue. For MEN1 surveillance and screening, some clinical guidelines generally advise the anatomical localization of NETs in conjunction with clinical characteristics and biochemical results.^[8,9]^ However, there is little data and a lack of consensus guidelines^[10]^ on the most precise methods for screening patients with MEN1-related tumors, and patients may present with metastases as soon as they are diagnosed.

A traditional imaging technique is used to detect and offer anatomical localization and staging of a tumor before surgery, CT scans provides a wider view of tumor morphology, location, and extent of the tumor, whereas MR images with contrast enhancement can offer a better view of blood flow, anatomy, and diffusion restriction.^[11]^ Previous studies^[12,13]^ have shown that approximately 10% of pNET have multiple insulinomas, which are generally associated with MEN1 syndrome in the meantime. In general, it is difficult to locate small MEN1-associated tumors and to depict specific endocrine characteristics.

The distinctive increase in the incidence of NETs has been ascribed to improved diagnostic and pathological techniques over the last few decades. Compared with other conventional imaging patterns, functional imaging is a noninvasive imaging technique that distinguishes most insulinomas. Studies have reported ^68^Ga-exendin-4 PET/CT as a valuable and credible imaging technique to distinguish MEN1-associated insulinomas. In the detection of MEN1-associated benign insulinomas, the sensitivity of PET/CT was 84.6%^[14]^ because of glucagon-like peptide-1 (GLP-1) receptors are highly expressed in benign insulinomas.^[14–16]^ Similarly, Sowa-Staszczak et al^[17]^ reported that the sensitivity and specificity of GLP-1 receptor imaging are 100% in patients with benign insulinomas. There have been several studies on the diagnostic performance of ^68^Ga-DOTATATE PET/CT in patients with MEN1, and it is available for detecting MEN1-associated tumors.^[18–20]^ For insulinomas, it is well established that SSTR2 densities are lower than other types of pNETs, which could, in combination with the small size of the lesion, lead to false-negative findings during SSTR imaging.

Wild et al^[21]^ showed that compared with benign insulinomas, the majority of malignant insulinomas often lack GLP-1 receptors but are more likely to express SSTR2 receptors. The Previous studies^[22,23]^ showed that only 36% of the malignant insulinomas expressed GLP-1 receptors, when compared to benign insulinomas. Zimmer et al^[24]^ reported SSTR scintigraphy showed low detection efficiency (<20%) in benign insulinomas and a higher positive rate in 73% of malignant insulinomas. Recent study^[25]^ has found that ^68^Ga-DOTATATE PET/CT provides better identification of insulinomas (9/10, 90%) in comparison with other imaging modalities, 8 of 9 tumors had Ki-67 of <2%, the diameter of insulinoma is about 0.7 to 2.5 cm, but sensitivity and accuracy of ^68^Ga-DOTATATE were not mentioned in benign and malignant insulinomas, may be too little concerned with the number of cases.

Our case demonstrates MEN1-associated low-grade insulinomas along with higher ^68^Ga-DOTATATE tracer uptake, which is significant in the proper diagnosis of MEN1-associated low-grade insulinomas with SSTR expression. Pituitary MRI revealed a pituitary tumor with mild ^68^Ga-DOTATATE uptake, which may be related to small lesions and decreased SSTR2 expression. Especially remarkable is that serum PTH was abnormally high in this patient, whereas ^68^Ga-DOTATATE and ^18^F-FDG PET/CT parathyroid imaging showed no abnormality in the parathyroid region, probably due to the small size of the lesion at present; adenomas of the parathyroid should be watched carefully over her lifetime. For this patient, the treatment focused on insulinoma derived from the pancreatic uncinate process, and surgical resection was the preferred treatment choice. The patient’s hypoglycemic symptoms disappeared after surgical resection.

Kornaczewski Jackson et al^[26]^ proposed that ^18^F-FDG PET/CT could be helpful for MEN1 patients with pNETs with a higher malignant potential. We conducted a ^68^Ga-DOTATATE and ^18^F-FDG PET/CT scan, and the imaging results showed no apparent abnormal ^18^F-FDG uptake in the pancreatic uncinate process; thus, the possibility of MEN1-associated malignant insulinoma may be very small, and immunohistochemistry eventually confirmed this result.

In conclusion, we present a rare case of MEN1-associated tumors with low-grade insulinoma and parathyroid and pituitary tumors, which showed MEN1 associated low-grade insulinomas along with higher ^68^Ga-DOTATATE tracer uptake. ^68^Ga-DOTATATE PET/CT imaging may be an available nuclear imaging tool for the detection of MEN1-related tumors and preoperative localization of small and low-grade insulinomas by PET/CT.

## Author contributions

**Conceptualization:** Yunuan Liu, Xinming Zhao, Jingmian Zhang, Jianfang Wang.

**Data curation:** Yunuan Liu, Zhaoqi Zhang, Meng Dai.

**Investigation:** Yunuan Liu, Meng Dai, Na Wang, Fenglian Jing, Tingting Wang, Weiwei Tian.

**Supervision:** Xinming Zhao, Jingmian Zhang, Jianfang Wang.

**Writing – original draft:** Yunuan Liu.

**Writing – review & editing:** Xinming Zhao.
